# The extent of rapid colour change in male agamid lizards is unrelated to overall sexual dichromatism

**DOI:** 10.1002/ece3.10293

**Published:** 2023-07-09

**Authors:** Anuradha Batabyal, Amod Zambre, Tess Mclaren, Katrina J. Rankin, Ruchira Somaweera, Devi Stuart‐Fox, Maria Thaker

**Affiliations:** ^1^ Department of Physical and Natural Sciences FLAME University Pune India; ^2^ Centre for Ecological Sciences Indian Institute of Science Bengaluru India; ^3^ Department of Ecology, Evolution and Behavior University of Minnesota Minneapolis Minnesota USA; ^4^ School of BioSciences The University of Melbourne Parkville Victoria Australia; ^5^ Stantec Australia Perth Western Australia Australia; ^6^ School of Biological Sciences University of Western Australia Perth Western Australia Australia

**Keywords:** chromatophore, physiological colour change, reptile, sexual dichromatism

## Abstract

Dynamic colour change is widespread in ectothermic animals, but has primarily been studied in the context of background matching. For most species, we lack quantitative data on the extent of colour change across different contexts. It is also unclear whether and how colour change varies across body regions, and how overall sexual dichromatism relates to the extent of individual colour change. In this study, we obtained reflectance measures in response to different stimuli for males and females of six species of agamid lizards (Agamidae, sister family to Chameleonidae) comprising three closely related species pairs. We computed the colour volume in a lizard‐vision colour space occupied by males and females of each species and estimated overall sexual dichromatism based on the area of non‐overlapping male and female colour volumes. As expected, males had larger colour volumes than females, but the extent of colour change in males differed between species and between body regions. Notably, species that were most sexually dichromatic were not necessarily those in which males showed the greatest individual colour change. Our results indicate that the extent of colour change is independent of the degree of sexual dichromatism and demonstrate that colour change on different body regions can vary substantially even between pairs of closely related species.

## Graphical abstract

We studied the extent of dynamic colour change in both sexes in six agamid species. This study indicates that the extent of colour change is independent of the degree of sexual dichromatism and demonstrates that colour change on different body regions can vary substantially even between pairs of closely related species.

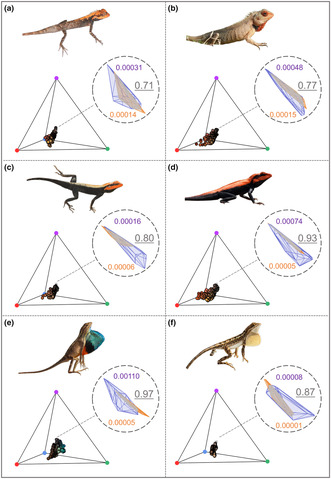

## 1 INTRODUCTION

Dynamic colour change is widespread in ectothermic animals and enables individuals to accommodate competing selection pressures on colouration (Stuart‐Fox & Moussalli, 2009). For example, chameleons are highly cryptic, changing colour to match different backgrounds, but also dynamically show conspicuous colours in response to conspecifics (Stuart‐Fox & Moussalli, 2008). Dynamic colour change, also known as physiological colour change, involves movement of pigments within the integument on the temporal scale of seconds to minutes. This contrasts with morphological colour change, which involves changes in the quantity or composition of pigments and occurs on the temporal scale of days to months (Duarte et al., 2017; Stuart‐Fox & Moussalli, 2009). Dynamic colour change has been widely studied in the context of background matching and camouflage more generally (Caro et al., 2016; Stevens, 2016); however, colour‐changing animals may show different colour states depending on a wide range of contexts including different backgrounds, stress/physiological state, different predators, temperature and conspecifics. Currently, quantitative data on the extent of colour change across contexts exists for few species (but see Cadena et al., 2017, Carter et al., 2020, Duarte et al., 2017, Stevens et al., 2014, Teyssier et al., 2015). Additionally, most studies focus on individual species; it is only by comparing colour change in multiple related species that we can begin to ask questions about similarities and differences in the ecological and evolutionary drivers of colour change among related and independent lineages.

For animals that use colour signals for intra‐specific communication, sexual selection can result in sexual dichromatism by favouring more conspicuous dynamic or static colours in one sex, usually males. For example, in chameleons, species that are more sexually dichromatic are also those in which males change colour the most, suggesting that sexual selection has driven the evolution of male colour change (Hutton et al., 2015; Ligon & McGraw, 2018; Stuart‐Fox & Moussalli, 2008, 2009). By contrast, in many other groups of lizards, such as iguanids, males are usually more colourful than females but colour signals are largely static, showing limited or no dynamic change (Cooper & Greenberg, 1992). In colour‐changing species, it can be difficult to estimate sexual dichromatism because sexual dichromatism must account for the range of colours exhibited by both males and females. Although males and females may exhibit different colours at any given instance, there may be little overall sexual dichromatism if both sexes exhibit a similar dynamic range of colours (e.g. Dickerson et al., 2020). Thus, it remains unclear whether or how sexual dichromatism and the extent of individual colour change covary in colour‐changing species.

The extent of colour change often differs between body regions because different body regions are under different selection pressures (Stuart‐Fox & Moussalli, 2008, 2009). For example, bearded dragon lizards (*Pogona vitticeps*) show greater dorsal colour change for camouflage and thermoregulation requirements and greater throat colour change for signalling (Smith et al., 2016). By contrast, dorsolateral colour change in another agamid the Indian rock agama, *Psammophilus dorsalis*, differs depending on social context (Batabyal & Thaker, 2017). Males of *P. dorsalis* have distinct dorsal and lateral bands that change to different colours at different speeds in response to conspecific females and males (Batabyal & Thaker, 2017). Similarly, species with different ecologies and signalling requirements often differ in the body regions used for conspecific signalling (Stuart‐Fox & Ord, 2004). For example, many terrestrial lizards signal to conspecifics with colourful ventral patches (Assis et al., 2020; Langkilde & Boronow, 2010; Weiss, 2002; Whiting, 1999); whereas arboreal lizards often signal with conspicuous lateral colour patches, and some species of both terrestrial and arboreal lizards have evolved colourful dewlaps that they can extend or retract (Nicholson et al., 2007). Lastly, different components of colour change may be important for different functions; for example, luminance change is most relevant for thermoregulation; whereas both luminance and chromatic change may be important for signalling (Stuart‐Fox & Moussalli, 2009). A useful approach to understanding the different functions and ecological drivers of colour and colour change is to compare the extent and nature of colour change in closely related species that are biologically similar in other respects; however, few such comparative studies have been attempted.

Here, we quantify colour change in six species comprising three closely related species pairs of agamid lizards. The Agamidae is the sister family to chameleons and show dramatic colour change that rivals the speed and degree of chromatic change seen in chameleons (Batabyal & Thaker, 2017; Davis et al., 2022). We examine the relationship between sexual dichromatism and colour change and compare the extent of colour change among body regions and species that differ in their ecology. We chose three pairs of species found in southwest India: *Calotes versicolor* (*Calotes vultuosus*: Gowande et al., 2021) and *Monilesaurus rouxii* (Pal et al., 2018): (formerly *Calotes rouxii*; hence we refer to the *Calotes* species pair for brevity), *Sitana laticeps* and *Sarada darwini*, and *Psammophilus dorsalis* and *Psammophilus blanfordanus*. The three species pairs differ in body size, ecology and body regions used for signalling (summarised Table 1; qualitative details on colouration is provided below). We used three forms of standardised stimuli to elicit colour changes – temperature, social interactions and stress. First, we quantified colour volumes of males and females of each species under these stimuli and examined the area of non‐overlapping colour volume as an indicator of sexual dichromatism. Focusing on males, we then examined maximum individual colour change on different body regions and examined differences within and between species pairs in the extent of male colour change on three body regions: head, chest and throat and dorsal and lateral. We then tested for a relationship between sexual dichromatism and the average extent of male colour change. Together, these data lay the foundation for understanding selection for dynamic colour change in this group.

**TABLE 1:** Morphological and ecological characteristics of the study species.

	Species	Ecology	Body size (SVL)	Sexual dimorphism
*Calotes* species pair	*Calotes versicolor*	Arboreal	Male: 90–180 mm Female: 76–118 mm	Body size, colouration
	*Monilesaurus rouxii*	Arboreal	Male: 77 mm Female: 71 mm	Body size, colouration
*Sitana/Sarada* species pair	*Sitana laticeps*	Terrestrial	Male: 42–49 mm Female: 43–56 mm	Dewlap size
	*Sarada darwini*	Terrestrial	Male: 52.9–63.4 mm Female: 41–52 mm	Body size, dewlap size, colouration
*Psammophilus* species pair	*Psammophilus dorsalis*	Terrestrial	Male: 136 mm Female: 110 mm	Body size, colouration
	*Psammophilus blanfordanus*	Terrestrial	Male: 104 mm Female: 71 mm	Body size, colouration

## 2 MATERIALS AND METHODS

### 2.1 Agamid species

#### 2.1.1 Calotes species pair

**FIGURE 1:**
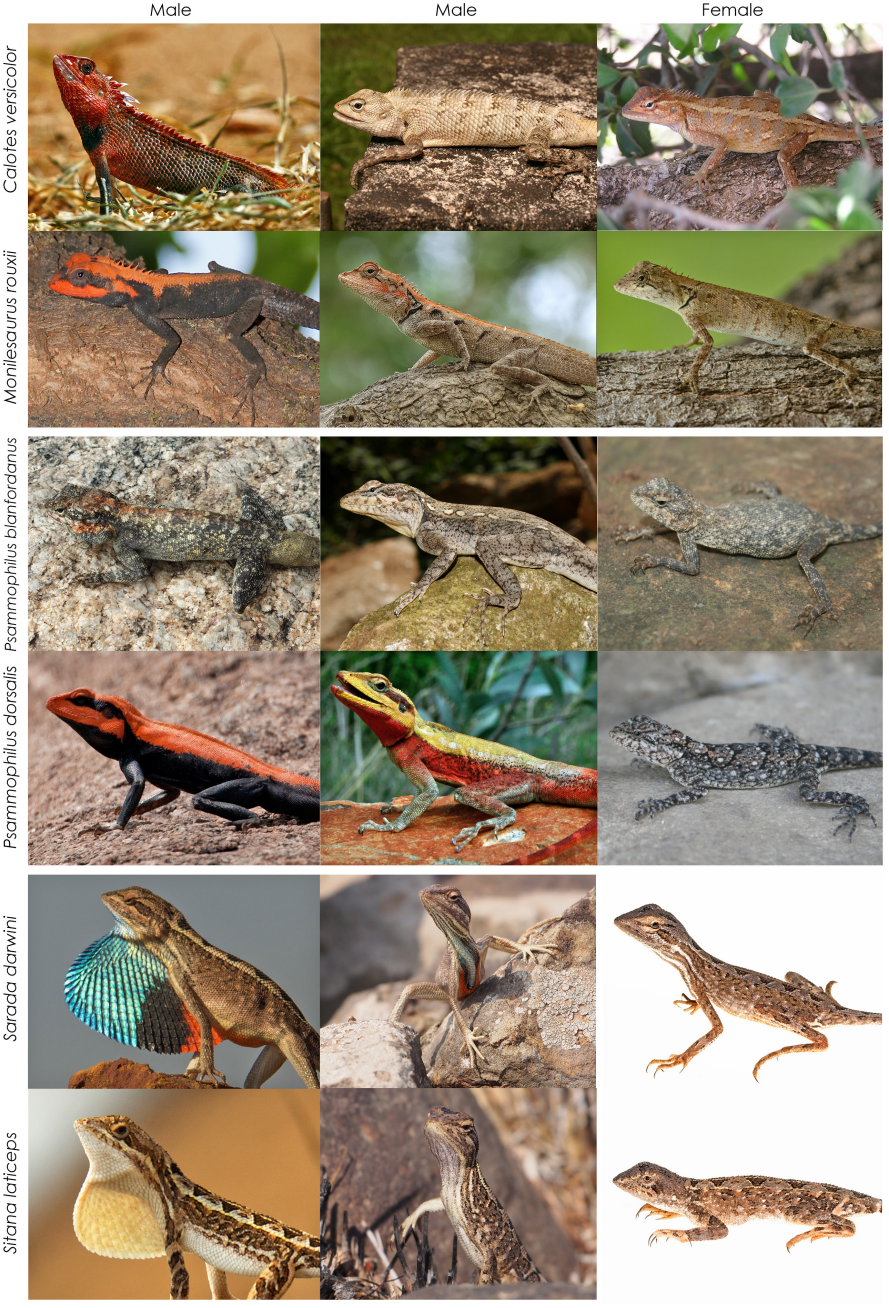
Images of each sex of each of the six species with examples of the different male colour states. Not all colour states are shown.

Both species (Figure 1) are primarily arboreal and exhibit rapid and marked colour change (Sreekar et al., 2011). *Calotes versicolor* is widespread throughout Asia and is a generalist species that has readily acclimatised to disturbed habitats (Radder, 2006). There is some variation in colouration and sexual dichromatism across populations, with males of *C. versicolor* showing more pronounced colour changes than females (A. Zambre, personal observation). Both sexes have a black patch at the neck region on the ventral side that signifies sexual maturity. During courtship, the head, throat, anterodorsal region and crest of males turn red (colour change starts within a minute and then lasts till courtship lasts). This change in colouration is the first step towards initiating courtship, and colour intensifies as courtship progresses (Pandav et al., 2007; A. Zambre, personal observation). At the end of the courtship display (lasting ~15–20 min), males return to a muted shade of olive green or brown (A. Zambre, personal observation). *Monilesaurus rouxii* is endemic to western India and inhabits a range of forested habitats (Sreekar et al., 2011). During the breeding season, males have black dorsolateral and ventral colouration along with a brilliant red head and crest whereas females have a reddish‐orange throat and a slaty black body. Both sexes anecdotally exhibit rapid colour change but this ability may be limited to the breeding season as during the non‐breeding season both sexes are olive green and the sexes are difficult to distinguish (Sreekar et al., 2011). During the non‐breeding season, both sexes exhibit dorsal darkening and lightening for background matching (A. Zambre, personal observation).

#### 2.1.2 Sarada and Sitana species pair

The fan‐throated lizards, *Sarada darwini* and *Sitana laticeps* (Figure 1) are both small terrestrial species that inhabit open rocky habitats of western India (Deepak et al., 2016; Zambre et al., 2020). Of these, *S. darwini* typically inhabits the foothills of the Western Ghats mountain range whereas *S. laticeps* are more widely distributed in the Deccan plateau regions. These species do, however, overlap in regions where the Western Ghats meet the Deccan plateau in western India. Males of both species have large dewlaps that are rapidly flagged during the breeding season for both intra and intersexual signalling (Zambre et al., 2020). There are, however, marked differences in the dewlap colouration between species; males of *S. laticeps* have a white dewlap whereas those of *S. darwini* have a bright tricolour dewlap consisting of blue, black and orange colours. Females of both species look remarkably similar and lack any bright colouration, with rudimentary white gulars and brownish body (Zambre et al., 2020). Although both species are known to use exaggerated dewlap displays during signalling, little is known of their colour change abilities except some change in tail colour to blue during intrasexual aggressive interactions among males (Deepak et al., 2016; Zambre & Thaker, 2017).

#### 2.1.3 *Psammophilus* species pair

*Psammophilus dorsalis* and *P. blanfordanus* (Figure 1) are large terrestrial lizards that occupy rocky habitat across southern and central India, respectively (Radder et al., 2006). *Psammophilus dorsalis* shows marked sexual dichromatism and rapid colour change for signalling (starts within seconds). Colour change in males is more pronounced and the dorsal and lateral body regions change colour independently during courtship and aggressive displays. Males change their dorsal colour to bright red and ventrolateral body to black when courting females and shift to a yellow dorsal colour and bright orange ventrolateral colour while displaying aggression (Batabyal & Thaker, 2017). Under neutral conditions males display a dull yellow dorsal and brown lateral body colouration. Females are generally patchy brown in colour and well camouflaged against the rocky background but turn their bodies darker and their heads red during some social encounters (Ranade et al., 2022). In contrast, males of *P. blanfordanus* show similar colour changes during both courtship and aggressive displays where they turn from a neutral dull brown to red colour in their head and crest region along with a dull black body. Females of *P. blanfordanus* are dull brown or olive green in colouration, turning their body darker and head a tinge of orange‐red when sexually receptive (A. Batabyal, personal observation).

#### 2.1.4 Study sites

**FIGURE 2:**
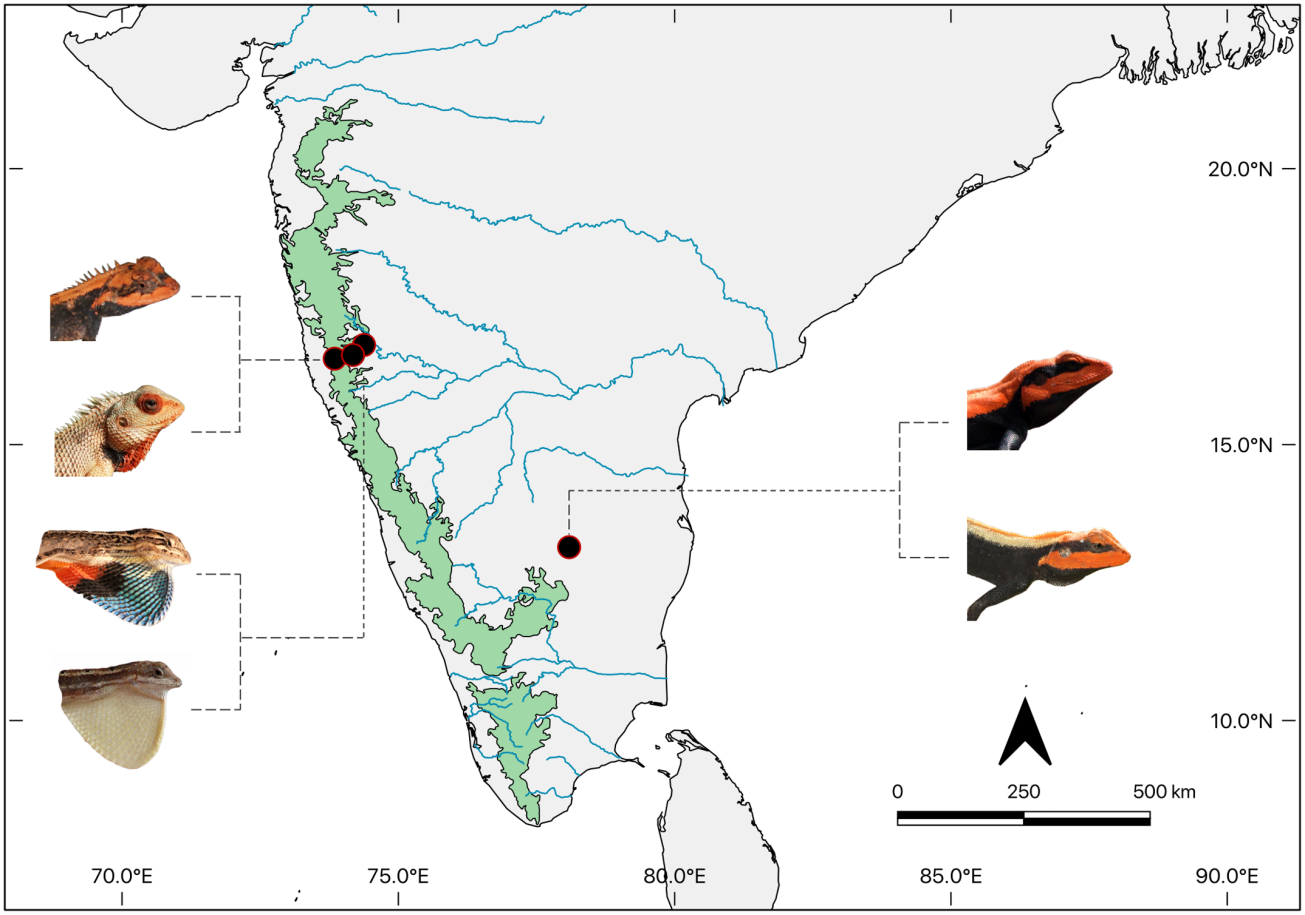
Collection localities of each of the six species. Images are of males of each species.

Lizards were captured either by hand or noosing poles in May–June 2016 from sites across southern and western India (Figure 2). *Calotes* species were collected along rural roadsides, bordering scrub and agricultural land, around the small village of Gaganbavda (16°33′10.9″N 73°50′45.2″E), near Kolhapur in western India. *Sarada darwini* were caught in Kagal (16°36′56.9″N 74°17′58.9″E) and Katyani (16°37′28.4″N 74°11′43.5″E) on dry rocky grasslands just outside the city of Kolhapur, India. *Sitana laticeps* were also caught just outside Kolhapur, in Kagal (16°36′56.9″N 74°17′58.9″E) and Wadgaon (16°47′44.8″N 74°22′45.5″E) on open rocky plateaus. *Psammopilus* species were caught on a sparsely scrubbed, rocky outcrop site with large boulders in the Kolar district, north of Bangalore, India (13°08′25″N 78°05′48″E).

#### 2.1.5 Data collection

For each species, we aimed to collect data for at least four females and six males; however, we were only able to collect data for three females of *P. dorsalis*. Sample sizes were: *Calotes versicolor* (CV): 4 females (F), 11 males (M); *M. rouxii* (MR): 4F, 11M; *Sarada darwini* (SD): 4F, 6M; *Sitana laticeps* (SL): 4F, 6M; *Psammophilus dorsalis* (PD) 3F, 9M; *P. blanfordanus* (PB): 4F, 6M. Captured lizards were kept in cloth bags for up to 5 days for the duration of the experiments and then released at the site of capture. During this time, the lizards were checked and misted with water daily.

We obtained reflectance measurements of lizards in response to the different stimuli described below. Colour change is a measure of the difference in colour between two states. In dynamically changing species we can only reliably measure colour in response to specific stimuli. We did not measure colour change as the difference in colour before and after exposure to a stimulus because there is no meaningful ‘baseline state’. The stimuli chosen, however, span a range of contexts and induce different forms and direction of colour change in agamids.

##### Temperature: 20 and 35°C

Lizards were placed in a temperature‐controlled incubator (32 cm L 36 cm W 45 cm H; Exo Terra, Rolf C. Hagen Corp.) at 20 and 35°C for 10 min at each temperature. These temperatures were selected because they capture the typical climatic range experienced by these species in the wild (Radder, 2006). We controlled for order effects in these experiments by conducting hot then cold temperature trials on half the individuals and the reverse order for the remaining half. After 10 min, lizards were removed from the incubator and reflectance measurements were quickly taken.

##### Conspecifics: males and females

We placed either two males or a male and a female into an open‐topped rectangular container (55 cm L 40 cm W 40 cm H; *Psammophilus* and *Calotes*, 40 cm L 35 cm W 20 cm H; *Sitana* and *Sarada*). These trials were observed from a location where the researchers could not be seen by lizards, for approximately 10 min or until colour change, physical aggression or courtship behaviour was observed. At this point, we quickly caught the focal lizard and measured reflectance.

##### Stress and early morning

Lizards were kept in cloth bags overnight and were often intensely coloured first thing in the morning; therefore, we measured individuals at 7 am, immediately after removing them from the bag. Moreover, we often observed the greatest colour change in response to handling stress so we measured individuals again after 1 min of handling. Additionally, where possible, we measured reflectance within 30 s of capture and again after 1 min of handling. This period of handling was sufficient to observe marked colour change.

**FIGURE 3:**
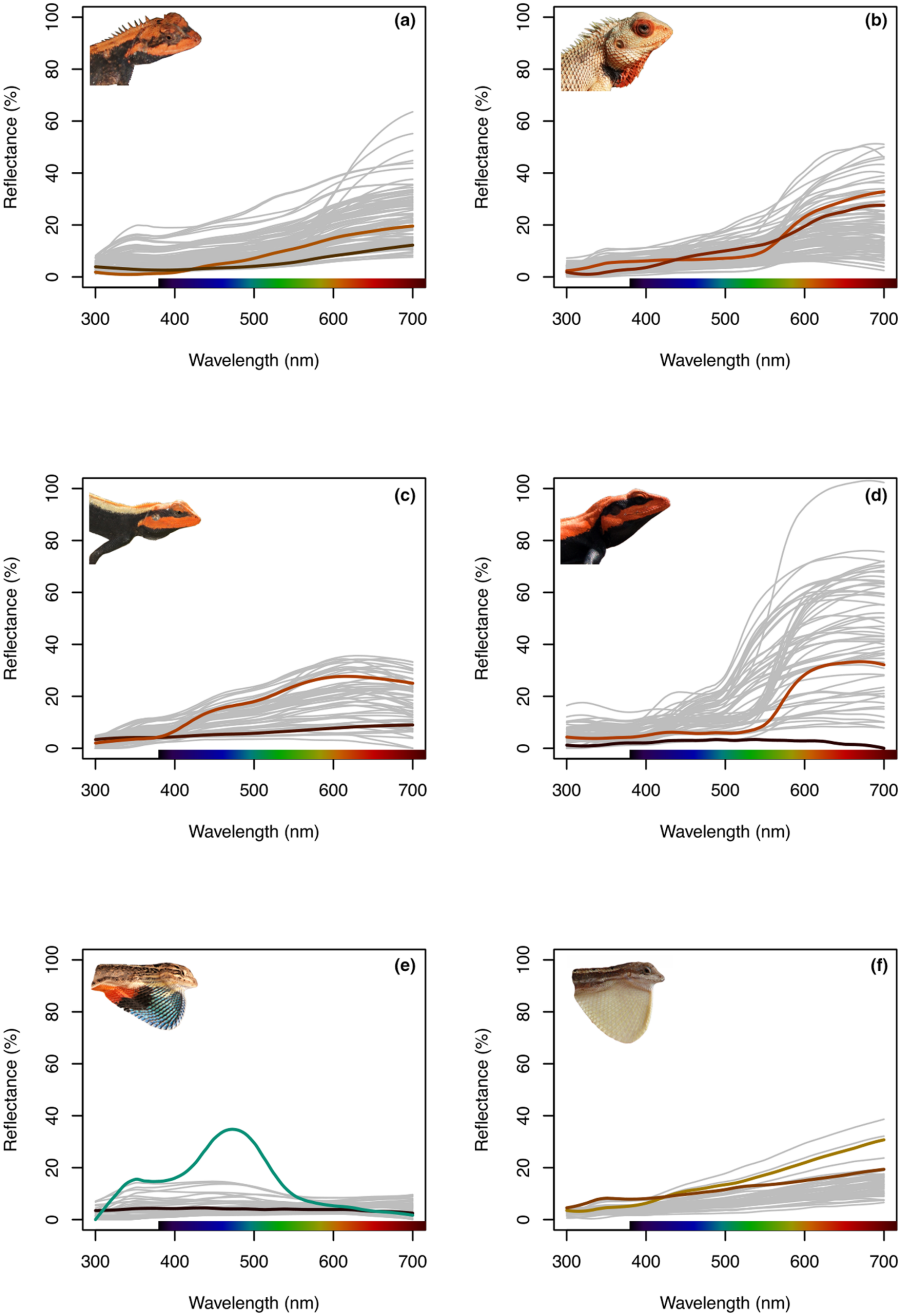
Variation in reflectance spectra for males of each species. Highlighted spectra show the maximum colour change (either chromatic or achromatic contrast) for a male for a specific body location. (a) *Monilesaurus rouxii*: chest/throat (b) *Calotes versicolor*: chest/throat (c) *Psammophilus blanfordanus*: chest/throat (d) *Psammophilus dorsalis*: dorsolateral (e) *Sarada darwini*: head and (f) *Sitana laticeps*: head.

We were unable to obtain reliable reflectance measurements for all individuals in all circumstances because some individuals did not show any behavioural or colour responses in some contexts. Females of *M. rouxii*, *P. dorsalis* and *S. laticeps* did not respond behaviourally or by changing colour under social context but males of all species responded under all contexts (though not every individual responded in all contexts). This enabled us to obtain sufficient spectra to estimate colour change for each individual and body region (females: mean = 4 spectra, range 2–6; males: mean = 4 spectra, range 2–7 spectra per body region per individual). Estimates of colour change from these spectra (Figure 3; details below) are likely to be an underestimate of the degree of change that an individual could exhibit; however, these measures of colour change enable comparison between body regions and species (but not between contexts).

Each species has different colour patterns and showed colour change on different specific body locations; therefore, we measured the reflectance of the specific locations relevant to each species (Figure S1). The locations measured for each species pair were: *Calotes* pair: mid‐dorsal, anterio‐lateral black blotch, mid‐lateral, crest, top of head, lip/side of head, gular flap, chest; *Sarada‐Sitana* pair: mid‐dorsal, crest, top of head, dewlap, chin; *Psammophilus* pair: mid‐dorsal, mid‐lateral, top of head, lip/side of head, throat. These were grouped into three broader regions to enable comparison among species pairs. The three body region categories were: (1) head, including crest and lip; (2) chest and throat (CT) including the gular flap and dewlap; (3) Dorsolateral (DL) including dorsal and lateral markings.

Lizard skin reflectance of each body region was measured in rapid succession using a JAZ EL‐200 spectrometer with an inbuilt Jaz PX pulsed xenon light source (Ocean Optics). The spectrometer was calibrated with a 99% white diffuse reflectance standard (WS‐1‐SL, Ocean Optics). The probe was fixed at 45° within a probe holder (either RPH‐1, Ocean Optics or custom‐made probe holder for smaller species) and placed against the lizard's skin to ensure consistent measurement distance and area (~4 mm^2^).

### 2.2 Data processing and analysis

We processed reflectance spectra using the R package pavo 2.0 (Maia et al., 2019). We visualised and quality‐checked all spectra, adjusted very minor negative reflectance values caused by electrical noise by lifting the curves by the maximum negative value, and then smoothed all spectra with LOESS smoothing of 0.16 using the procspec function.

We plotted spectra in tetrahedral colour space, where the four vertices are defined by the maximum quantum catch of each of the four photoreceptors of a tetrachromatic receiver, since diurnal lizards, including agamids, are tetrachromats. To calculate quantum catch, we used standard daylight irradiance (D65) in pavo 2.0 and spectral sensitivities based on the agamid lizard, *Ctenophorus ornatus* (Barbour et al., 2002) and *C. decresii* visual systems (Yewers et al., 2015): UVS *l*_max_ = 365 nm, SWS *l*_max_ = 440 nm, MWS *l*_max_ = 493 nm and LWS *l*_max_ = 571 nm. The relevant receiver—and indeed whether there is a receiver at all—depends on the context. We used a tetrachromatic lizard visual system. The absolute values of these estimates will differ slightly from those using a receiver‐independent colour volume (e.g. one based on hue, chroma and intensity) or using other tetrachromatic visual systems (e.g. birds), but the qualitative conclusions regarding sexual dichromatism and male colour change are unlikely to be affected.

#### 2.2.1 Colour volume and sexual dichromatism

We calculated the concave volume occupied by males and females of each species in the tetrahedral colour space described above using in pavo 2.0 (Maia et al., 2019). We specified ‘type = alpha’ in the colour volume function, which calculates concave volume with default parameter values described in Gruson (2020). These colour volumes describe the region of colour space occupied by males or females of a species: they include spectra of all body regions for multiple individuals with spectra for each individual taken in multiple conditions; therefore, they account for between‐individual variation as well as within‐individual variation in space (body regions) and time (colour change).

To derive a measure of sexual dichromatism, we first computed colour volume overlap between males and females. Sexual dichromatism (SD) was then calculated as the overlap standardised by the sum of male and female colour volumes:

$$\mathrm{SD}=\left({\mathrm{CV}}_{M+F}-{\mathrm{CV}}_{\text{overlap}}\right)/{\mathrm{CV}}_{M+F}.$$

where CV_M+F_ is the total male + female colour volume, and CV_overlap_ is the volume of overlap between males and females.

#### 2.2.2 Extent of individual colour change in males

We estimated the maximum extent of colour change in individual males, for which we obtained sufficient sample sizes. We estimated the maximum extent of colour change for each individual for a given body region as the maximum contrast between any pair of spectra taken from the exact same location measured in different contexts (Figure 3). We computed chromatic contrast (dS) and luminance contrast (dL), using the Receptor Noise Limited (RNL) model of colour vision (Vorobyev et al., 1998; Vorobyev & Osorio, 1998). The RNL model measures contrast as Just Noticeable Differences (JNDs) where ∆S = 1 is the theoretical threshold of discrimination between two stimuli viewed simultaneously under ideal viewing conditions against natural backgrounds (brown rocky substrate for six sampled species), assuming that discrimination is limited only by photoreceptor noise (i.e. excluding any influence of higher‐level processing; Vorobyev et al., 2001). Under natural conditions, discrimination thresholds are likely to be >1 and vary depending on the species and conditions (Olsson et al., 2017; Osorio et al., 2017). For this reason, we use the commonly used threshold of 2 JND as a more realistic threshold of colour discrimination under natural viewing conditions (though this threshold is arbitrary unless measured empirically).

The RNL model uses receptor quantum catches (calculated as above), adapted to background radiance and then scaled based on photoreceptor noise, which is a function of the relative density of each photoreceptor type within the retina. We used the natural brown background spectrum as the adapting background. To calculate photoreceptor noise (*w*_i_), we used a standard value of 0.1 for noise in the LWS photoreceptor and then calculated noise for the other photoreceptor classes assuming a ratio of 1 UVS:1 SWS:3.5 MWS:6 LWS (Barbour et al., 2002; Yewers et al., 2015). Luminance contrast was calculated using the sensitivity function for the LWS photoreceptor, assuming *w*_i_ = 0.05. All formulae, applied using this agamid visual system, are described in detail in Klomp et al. (2016, 2017), McLean et al. (2014) and Yewers et al. (2016).

### 2.3 Statistical analysis

All data were analysed in R statistical software version 3.6.0.

#### 2.3.1 Colour volumes

**FIGURE 4:**
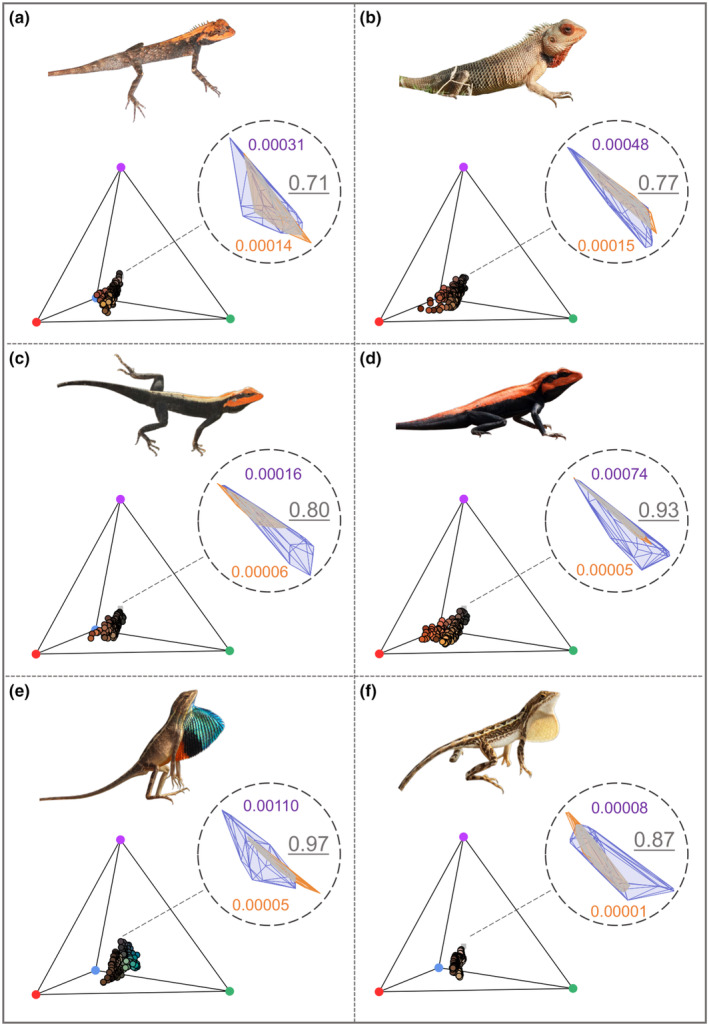
Colour volumes of males and females and sexual dichromatism for each of the six species. (a) *Monilesaurus rouxii* (b) *Calotes versicolor* (c) *Psammophilus blanfordanus* (d) *Psammophilus dorsalis* (e) *Sarada darwini* and (f) *Sitana laticeps*. Insets show values for male (purple) and female (orange) colour volumes as well as sexual dichromatism (value in large grey font) estimated from non‐overlapping male and female colour volumes, standardised by the sum of male and female colour volumes.

We computed colour volumes and sexual dichromatism as previously described. The area of non‐overlapping colour volumes in males and females (Figure 4) provides a visual representation of sexual dichromatism. We tested whether males were consistently more colourful than females across the six species by comparing the colour volume between sexes using the Wilcoxon test for non‐normal data. Our sample size for males is larger than for females. To exclude the possibility that males have larger colour volumes due to larger sample sizes, we also examined the correlation between colour volume and sample size (number of spectra making up the colour volume) using Pearson's correlation.

#### 2.3.2 Male colour change

We tested whether the extent of individual colour change in males differed between body regions and species using generalised linear mixed effects modelling using generalised estimating equations (package: geepack in R: Højsgaard et al., 2006). We used male chromatic or luminance change (maximum contrast between spectra across contexts for a given body region for each individual) as the response variable, and body region, species and their interaction as predictors. We also had individual ID as a random effect. We quantified the correlation structure between individual data points as ‘independence’ (i.e. response within subjects are not correlated) and ‘exchangeable’ (i.e. all pair of observations within a subject are equally correlated) and both models showed similar results along with the ‘Working Correlation Matrix’ that shows an estimate of 0 as the common correlation between pairs of observations within a subject thus allowing us to treat the ‘independence’ model in the result. The post hoc analysis for pairwise comparison was performed using emmeans package in R.

#### 2.3.3 Sexual dichromatism and male colour change

We examined the relationship between sexual dichromatism and male colour change. Our measure of sexual dichromatism is a species‐level measure (i.e. one value per species). To derive a species‐level measure of male colour change, we averaged maximum male chromatic change across all body regions and individuals for each species. We then examined the correlation between these species‐level measures of sexual dichromatism and male colour change using Pearson correlation.

## 3 RESULTS

### 3.1 Colour volumes

Males had significantly larger colour volume than females (Wilcoxon test: *W* = 2, *p* = .0086 Figure 4). Although the sample size was smaller for females than males, this effect was independent of sample size as we found no correlation between colour volumes and sample size (Pearson's correlation coefficient: *r* = .382, *p* = .2193, Figure S2).

These species differed in the extent of sexual dichromatism (Figure 4). The species with the greatest sexual dichromatism were *Sarada darwini* (sexual dichromatism, SD = 0.97) and *Psammophilus dorsalis* (PD = 0.93) and those with the lowest were *Calotes versicolor* (CV = 0.77) and *Monilesaurus rouxii* (MR = 0.71). *Sitana laticeps* (SL = 0.87) and *Psammophilus blanfordanus* (PB = 0.80) had intermediate sexual dichromatism.

Species also differed in male colour volumes with the most ‘colourful’ males in *S. darwini* (0.001) and least in *S. laticeps* (0.00008). Within species pairs, colour volume of males was higher in *P. dorsalis* than *P. blanfordanus*; marginally higher in *C. versicolor* than *M. rouxii* and higher in *S. darwini* than *S. laticeps* (Figure 4).

### 3.2 Colour change in males

**FIGURE 5:**
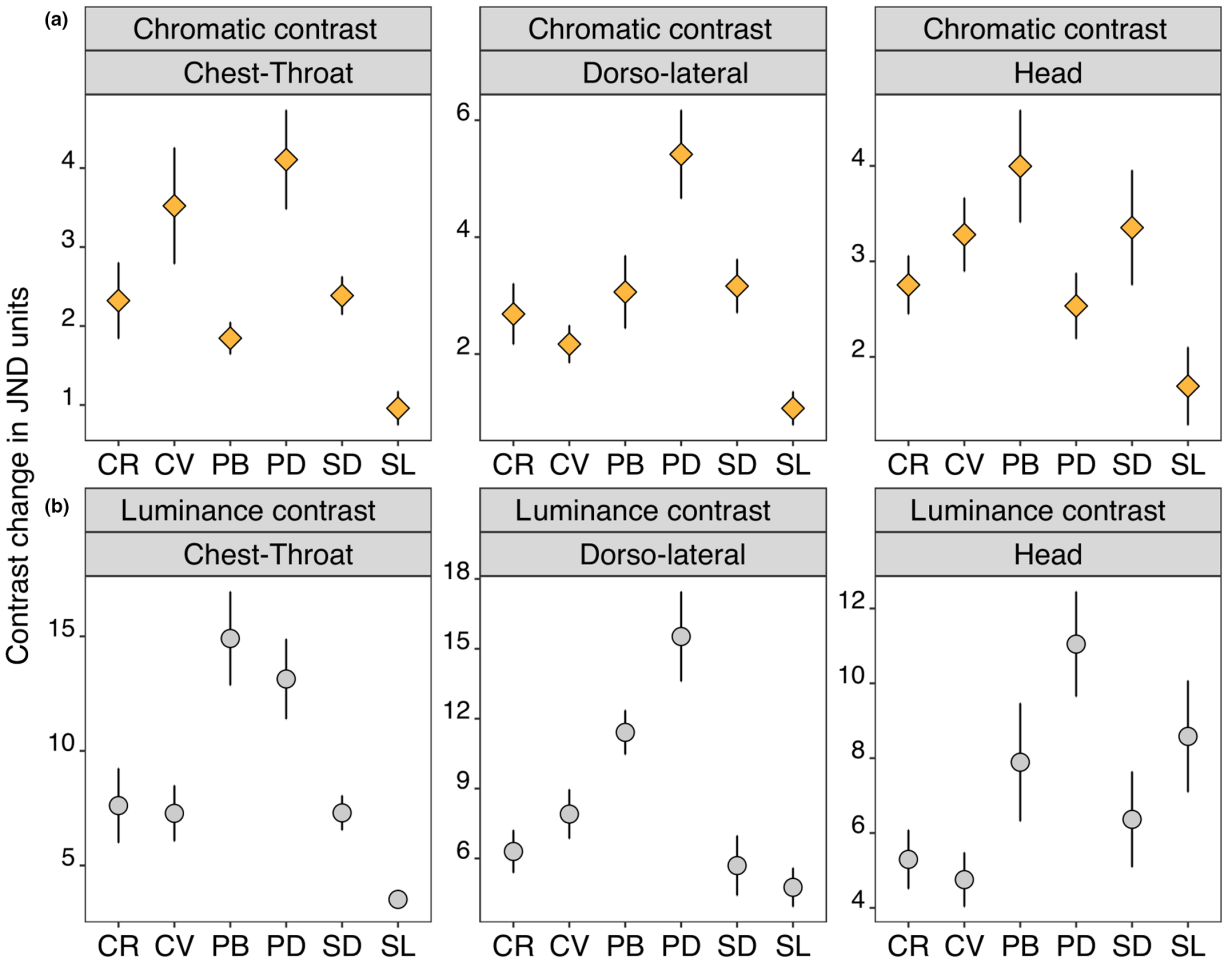
Maximum chromatic change (a. top row) and luminance change (b. bottom row) of males for each body region of each species (CV, *Calotes versicolor*; MR, *Monilesaurus rouxii*; PB, *Psammophilus blanfordanus*; PD, *Psammophilus dorsalis*; SL, *Sitana laticeps*; SD, *Sarada darwini*).

The species differed in male chromatic change, but this difference depended on body region (Anova Wald statistic: Species*body region *X* = 40.00, *p* < .001, Table 2). Within the *Psammophilus* species pair, chest‐throat colour change in *P. dorsalis* was higher than the corresponding body region in *P. blanfordianus* (*z* = 4.64, *p* < .001, Figure 5a). Chest‐throat chromatic change in *P. dorsalis* was higher than the change observed for the same region in *S. darwini* and *S. laticeps* (chest‐throat chromatic change: PD vs. SD: *z* = −3.39, *p* = .009; PD vs. SL: *z* = −3.73, *p* = .003; Figure 5a). Chromatic change in chest‐throat in male *S. darwini* was higher than that for the corresponding body region in *S. laticeps* (*z* = −2.90, *p* = .040). Chromatic change in *S. laticeps* was <2 JND for all three body regions indicating that any colour change was minor and unlikely to be noticeable by conspecifics. Chromatic change in the *Calotes* species pair was similar for all three body regions (all *p* > .05; Figure 5a).

**TABLE 2:** ANOVA tables reporting differences in (a) chromatic change and (b) luminance change between species, body region and the interaction between species and body region.

Predictor	df	X2	*p* Value
(a) Chromatic change			
Species	5	29.10	<.0001
Body region	2	0.80	.680
Species*Body region	10	40.00	<.001
(b) Luminance change			
Species	5	73.20	<.001
Body region	2	6.30	.042
Species*Body region	10	52.20	<.001

When considering male luminance change, we found a significant interaction between species and body region (Anova Wald statistic: Species*body region *X* = 52.20, *p* < .001, Table 2). Similar to chromatic contrast, luminance change in *P. dorsalis* was higher than in the other species, especially for the dorsolateral body region (dorsolateral luminance change: PD vs. MR: *z* = 4.09, *p* = .001; PD vs. CV: *z* = 3.51, *p* = .006; PD vs. SD: *z* = −3.03, *p* = .030; PD vs. SL: *z* = −4.27, *p* < .001; Figure 5b). However, within each species pair apart from chest‐throat contrast in *S. darwini* and *S. laticeps* there were no significant differences between males in luminance change for any body region (chest‐throat: SL vs. SD: *z* = −5.55, *p* < .001; all other *p* > .05; Figure 5b).

### 3.3 Sexual dichromatism and male colour change

There was no correlation between male colour volume and maximum chromatic change averaged across body regions and individuals for each species (correlation coefficient = −0.589, *t* = 1.46, df = 4, *p* = .218). Additionally, there was no correlation between our measure of sexual dichromatism and maximum chromatic change averaged across body regions and individuals for each species (Correlation coefficient = 0.194, *t* = 0.396, df = 4, *p* = .711; Figure S3).

## 4 DISCUSSION

Sexual selection has resulted in the evolution of sexual dichromatism in a wide range of species (Badyaev & Hill, 2003). Dichromatism is typically measured for individual body regions or as a composite measure for the whole body (Cally et al., 2021; Cooney et al., 2019; Portik et al., 2019; Stuart‐Fox & Ord, 2004; Wagner et al., 2012), but rarely, if ever, accounts for colour change, which is widespread in ectothermic vertebrates (Bell et al., 2017; Losos, 2011; Nilsson Sköld et al., 2013; Stuart‐Fox & Moussalli, 2009). The ability to change colour extends the colour space available to a species for communication, or other functions such as thermoregulation or camouflage. Colour volumes that incorporate rapid dynamic colour change provide an indication of the total colour space occupied by males or females of a species, and better captures the extent of sexual dichromatism. In this study, we captured the broadest range and extent of colour change in six species of agamid lizard. In all species, males have larger colour volumes than females; however, the area of non‐overlapping colour volumes differed, indicating species differences in the extent of sexual dichromatism. Surprisingly, the extent of sexual dichromatism did not seem to correspond to the extent of colour change in males. For example, *S. darwini* are sexually dichromatic as only males are vibrantly coloured and these colours occupy a large colour volume, yet colour change is limited. Thus, in contrast to chameleons (Ligon & McGraw, 2016; Stuart‐Fox & Moussalli, 2008), species that are most sexually dichromatic are not necessarily those in which males also change colour the most.

As expected from species with polygamous mating systems, males of these agamid species are more colourful (i.e. have larger colour volumes) than females. This pattern of sexual dichromatism is typical of lizards that also exhibit static colours that show minimal, if any, dynamic colour change over short time scales (Chen et al., 2012; Pérez i de Lanuza et al., 2013; Stuart‐Fox & Ord, 2004). The degree of sexual dichromatism, however, did not seem to predict the extent of male colour change. The modest sample size in this study may result in an underestimate of colour volume within species and colour change within males; however, it is unlikely to affect qualitative conclusions because the differences between body regions, sexes and species are comparatively large (and there was no significant correlation between colour volume and sample size). In contrast to the dynamically changing species in this study, sexual dichromatism in many lizard species with static colour signals is correlated with other forms of sexual dimorphism such as body size differences and behavioural displays. For example, male‐biased sexual dimorphism in body size is a significant predictor of sexual dichromatism in lacertids (Pérez i de Lanuza et al., 2013). In Australian agamids, colour pattern complexity of males is significantly associated with sexual dichromatism (Chen et al., 2012). In several iguanids, behavioural display complexity of males is positively correlated to sexual size dimorphism (Cox et al., 2003), which is also a trait associated with sexual selection similar to sexual dichromatism.

Dichromatism that is enhanced by colour change is not unique to agamids. Many frogs show dynamic dichromatism, induced only during the breeding season (Bell et al., 2017; Bell & Zamudio, 2012). In the agamid species we examined, there is no clear indication that sexual selection has driven the extent of colour change in the males of the species. Colour change increased male colour volume, particularly in *Psammophilus dorsalis*, but this had little impact on the extent of overlap with female colour volume, indicating that colour change is largely independent of sexual dichromatism in these species. The wide variety of colours in these species along with the range of colour‐changing abilities might result in the greater variation that we observe, thus making it difficult to discern any consistent pattern for the evolution of sexual dichromatism. Sexual dichromatism might be influenced by both natural and sexual selection in agamids and dichromatism in specific body regions might be associated with habitat characteristics (Stuart‐Fox & Ord, 2004). The interesting finding in our study is that related species pairs that occupy similar habitats also show large variations in colour volumes, colour change and sexual dichromatism. Thus, it provides us with a unique system in which to further investigate the drivers for the decoupling of sexual dichromatism and the extent of colour change in closely related species.

Males of all species except *Sitana laticeps* showed some degree of colour change. The colour change that was induced under the different socio‐ecological conditions was >2 JND for all body regions for all species except *S. laticeps*, suggesting that these changes are likely to be detectable by conspecifics. They are also likely to be seen by potential predators, such as tetrachromatic birds (Endler & Mielke, 2005). It is widely held that body regions exposed to visual predators should be less conspicuous (Endler, 1992; McLean et al., 2014; Stuart‐Fox & Ord, 2004); therefore, we expected the dorsal body region of these species to show the least colour change. However, in our data set, dorsolateral change was not notably less than for other body regions in the males of all species. In fact, dorsolateral change in *Psammophilus dorsalis* was greater than for any other body region in any other species. *Psammophilus dorsalis* males have distinct, wide, dorsal and lateral bands, both of which show marked colour change under different conditions. This conspicuous dorsal colours of *P. dorsalis* males, specifically the black lateral and red dorsal hue used during courtship interactions, is also risky as it is most conspicuous to different visual systems of predators and elicits the highest predator attack rates in the wild (Amdekar & Thaker, 2019). Although the conspicuous colours of *P. dorsalis* make it easily detectable to predators, the ability to rapidly change between different colour states allows the evolution of such social signalling colours that can be balanced against predation cost. A recent study in *Anolis aquaticus* also showed the advantages of rapid colour change to evade predators while moving across different microhabitats (Wuthrich et al., 2022).

Aside from the dorsolateral colour change in *P. dorsalis*, the chest‐throat region in *P. dorsalis* also showed significantly high chromatic contrast compared to the same body region in other species. The chest‐throat region in *S. darwini* showed higher chromatic and luminance contrast compared to its sister *S. laticeps*. Other than that there were no consistent differences in colour change of different body regions, even within species pairs. Instead, we observed variation in the combination of sexual dichromatism and male colour change. *Sarada darwini* males showed the greatest colour volume compared to other species but did not show a high degree of colour change. Thus, each species has evolved its own unique colour signalling wherein some use multiple static colours displayed across different body regions while others use rapid colour change to display multiple colours in the same body region. Closely related species often show similarities in their behaviour and visual display traits (Harvey & Pagel, 1991). However, when closely related species pairs occupy the same habitat or exist in sympatry the signals tend to diverge to enhance species recognition (Grether et al., 2009; Ptacek, 2000; Shizuka & Hudson, 2020). This might be one of the reasons why we observed a striking difference between *S. laticeps* and *S. darwini* where one occupies a much smaller colour volume than the other (Zambre et al., 2020). In the *Psammophilus* species pair, we observed *P. dorsalis* to have greater colour change (chest‐throat) compared to its related sister *P. blanfordanus*. However, the extent of colour and luminance change depended on the body region. In the *Psammophilus* pair, chromatic change of the chest‐throat but luminance change in dorsolateral regions were greater in *P. dorsalis* than *P. blanfordanus*. Divergence in signalling colours can also evolve if species use different microhabitats where the light environment is associated with signal detectability. Several examples of such signal divergence are found in lizards and birds (McNaught & Owens, 2002; Ng et al., 2013; Simpson et al., 2021). Incorporating microhabitat and lighting variation into future studies of colour change would allow for a broader understanding of the environmental drivers of colour signal divergence.

## 5 CONCLUSIONS AND FUTURE DIRECTIONS

Agamids are emerging as a lizard group that exhibits immense diversity in colour signals and colour change. Our results indicate that colour change on different body regions can vary substantially between sexes and even between pairs of closely related agamid lizard species. Although males change colour more than females and have larger colour volumes as a consequence, the degree of male colour change does not necessarily correspond to sexual dichromatism because the sexes can differ in colours that show little dynamic change. Thus, agamid lizards could be an interesting system to disentangle selection resulting in sexual dichromatism and dynamic colour change used in social or sexual signalling. The range of colours observed in the agamids includes structural and pigment‐based colours and this can serve as a model to investigate the mechanism and physiology of the various colour expression via molecular genetic approaches (genes involved in colour production and differential gene expression). A detailed phylogenetic study along with colour signalling traits, and microhabitat characteristics will also allow us to understand the evolution of rapid colour change in some lineages compared to others and the convergence and divergence of this trait along evolutionary history. Overall, this study system provides a basis for understanding the ecological and evolutionary drivers of colour change among related but independent lineages.

## AUTHOR CONTRIBUTIONS

**Anuradha Batabyal:** Formal analysis (equal); investigation (equal); methodology (equal); writing – original draft (equal); writing – review and editing (equal). **Amod Zambre:** Formal analysis (lead); investigation (equal); methodology (equal); writing – original draft (equal); writing – review and editing (equal). **Tess Mclaren:** Investigation (equal); methodology (equal); writing – review and editing (equal). **Katrina J. Rankin:** Investigation (equal); writing – review and editing (equal). **Ruchira Somaweera:** Supervision (supporting); writing – review and editing (equal). **Devi Stuart‐Fox:** Conceptualization (lead); funding acquisition (lead); investigation (equal); methodology (equal); supervision (lead); writing – original draft (equal); writing – review and editing (equal). **Maria Thaker:** Conceptualization (lead); funding acquisition (lead); investigation (equal); methodology (equal); supervision (lead); writing – original draft (equal); writing – review and editing (equal).

## CONFLICT OF INTEREST STATEMENT

The authors declare no competing interests.

## Supporting information


Appendix S1.

## Data Availability

The data sets used during the current study are available on Dryad: https://doi.org/10.5061/dryad.9cnp5hqq4
