# Supplementary material for: The extent of rapid colour change in male agamid lizards is unrelated to overall sexual dichromatism
Source: Ecol Evol. 2023 Jul 9;13(7):e10293. doi: 10.1002/ece3.10293 (PMC10329938; doi:10.1002/ece3.10293)
Supplement: Supplementary file 1 — Appendix S1. [file ECE3-13-e10293-s001.docx]

**The extent of rapid colour change in male agamid lizards is unrelated to overall sexual dichromatism**

Anuradha Batabyal ^1,,4#^ , Amod Zambre^2,4#^ , Tess Mclaren^3^, Katrina J. Rankin ^3^, Ruchira Somaweera^5,6^, Devi Stuart-Fox^3*^, Maria Thaker^4*^

**Affiliations**

^1^ Department of Physical and Natural Sciences, FLAME University, Pune, India

^2^ Department of Ecology, Evolution and Behavior, University of Minnesota

# joint first authors in alphabetical order

^3^ School of BioSciences, The University of Melbourne, Parkville, Victoria, Australia

^4^ Centre for Ecological Sciences, Indian Institute of Science, Bengaluru, India

^5^ Stantec Australia, Perth, Australia

^6^ School of Biological Sciences, University of Western Australia, Australia,

^*^Joint senior authors, in alphabetical order

**Corresponding author:** anuradha.batabyal@flame.edu.in

**Supplementary Information**

**
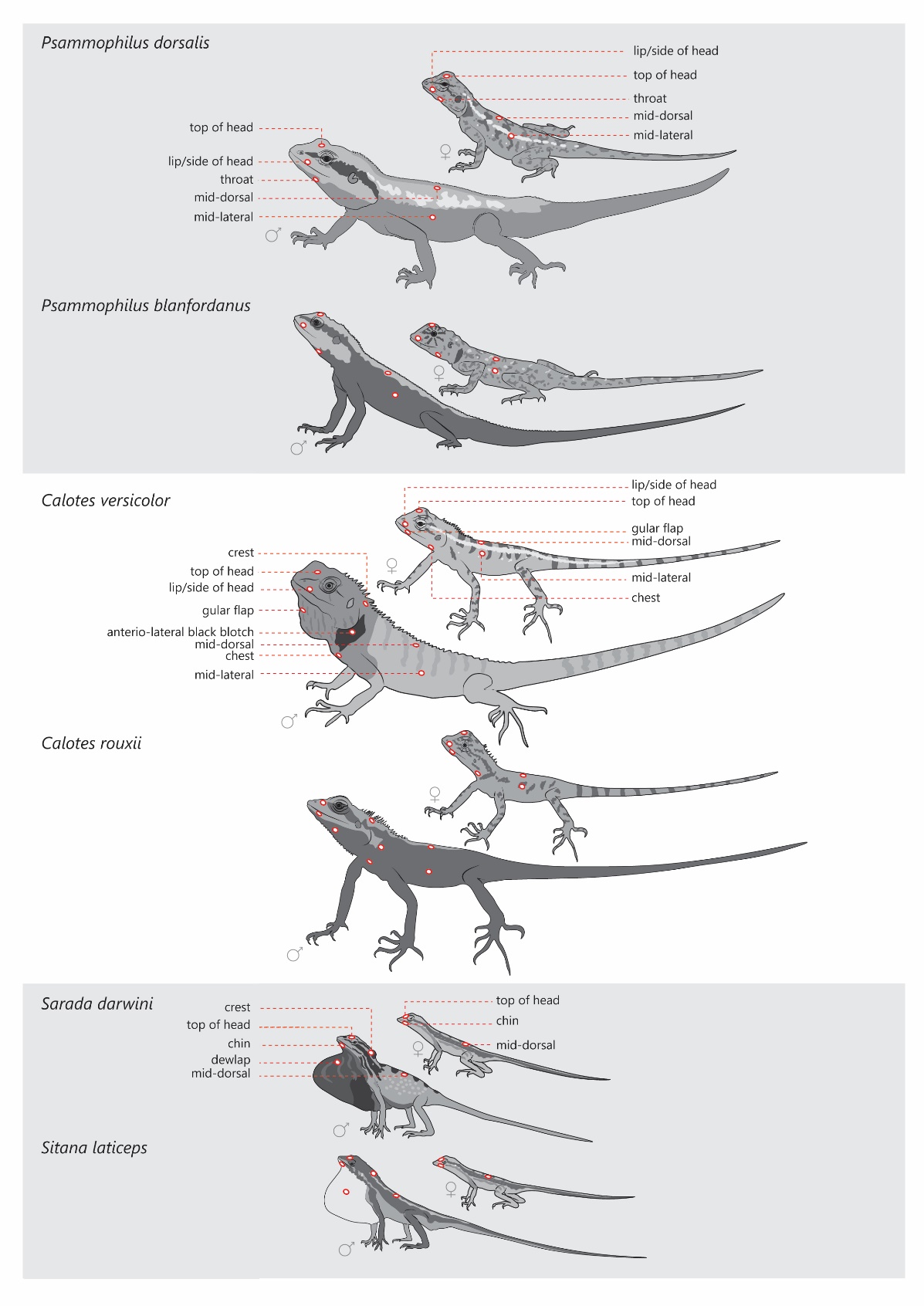
**

**Figure S1 -** Locations from which spectral measurements were taken, for each species. To enable comparison of body regions between species, the locations were grouped into three body regions: head, chest/throat and dorsal/lateral.


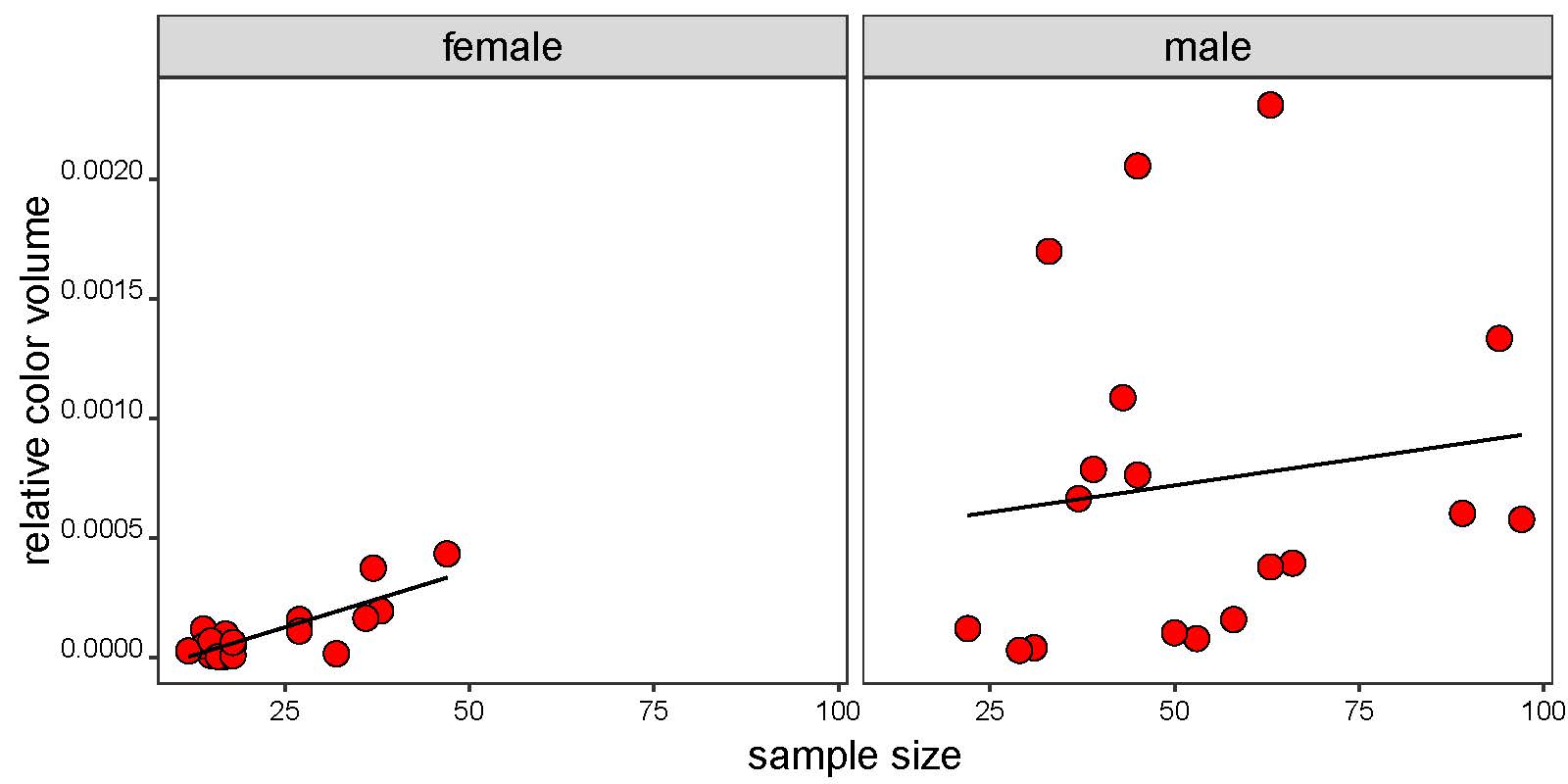


**Figure S2 –** Correlation of sample size (number of spectra) and relative colour volume in males and females. Each dot represents the spectra (colour volume and number of spectra used to calculate it) for a given body region of a given species.


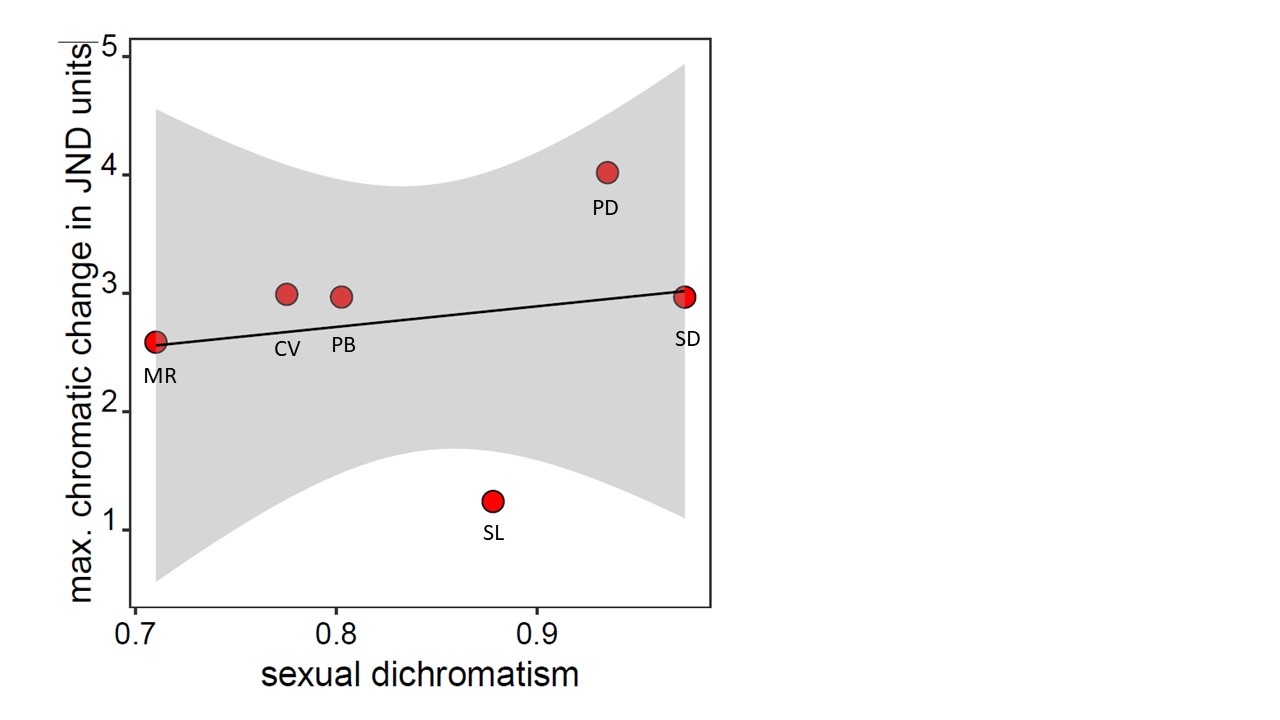


**Figure S3.** Correlation between sexual dichromatism of each species (CV: *Calotes versicolor*, MR: *Monilesaurus rouxii*, PB: *Psammophilus blanfordanus*, PD: *Psammophilus dorsalis*, SL: *Sitana laticeps*, SD: *Sarada darwini*) and the mean of the ‘maximum chromatic change’ averaged across body regions and individuals. Each data point corresponds to a species marked by respective acronyms.
